# Vitamin C and Infections

**DOI:** 10.3390/nu9040339

**Published:** 2017-03-29

**Authors:** Harri Hemilä

**Affiliations:** Department of Public Health, University of Helsinki, Helsinki FI-00014, Finland; harri.hemila@helsinki.fi; Tel.: +358-41-532-9987

**Keywords:** ascorbic acid, bacteria, bacterial toxins, common cold, herpes zoster, pneumonia, protozoa, respiratory tract infections, viruses, tetanus

## Abstract

In the early literature, vitamin C deficiency was associated with pneumonia. After its identification, a number of studies investigated the effects of vitamin C on diverse infections. A total of 148 animal studies indicated that vitamin C may alleviate or prevent infections caused by bacteria, viruses, and protozoa. The most extensively studied human infection is the common cold. Vitamin C administration does not decrease the average incidence of colds in the general population, yet it halved the number of colds in physically active people. Regularly administered vitamin C has shortened the duration of colds, indicating a biological effect. However, the role of vitamin C in common cold treatment is unclear. Two controlled trials found a statistically significant dose–response, for the duration of common cold symptoms, with up to 6–8 g/day of vitamin C. Thus, the negative findings of some therapeutic common cold studies might be explained by the low doses of 3–4 g/day of vitamin C. Three controlled trials found that vitamin C prevented pneumonia. Two controlled trials found a treatment benefit of vitamin C for pneumonia patients. One controlled trial reported treatment benefits for tetanus patients. The effects of vitamin C against infections should be investigated further.

## 1. Early History on Vitamin C and Infections

Vitamin C was identified in the early twentieth century in the search for a substance, the deficiency of which would cause scurvy [1,2]. Scurvy was associated with pneumonia in the early literature, which implies that the factor that cured scurvy might also have an effect on pneumonia. 

Alfred Hess (1920) summarized a series of autopsy findings as follows: “pneumonia, lobular or lobar, is one of the most frequent complications (of scurvy) and causes of death” and “secondary pneumonias, usually broncho-pneumonic in type, are of common occurrence and in many (scurvy) epidemics constitute the prevailing cause of death” [3]. He later commented that in “infantile scurvy ... a lack of the antiscorbutic factor (vitamin C) which leads to scurvy, at the same time predisposes to infections (particularly of the respiratory tract) ... Similar susceptibility to infections goes hand in hand with adult scurvy” [4]. In the early 1900s, Casimir Funk, who coined the word “vitamin”, noted that an epidemic of pneumonia in the Sudan disappeared when antiscorbutic (vitamin C-containing) treatment was given to the numerous cases of scurvy that appeared at about the same time [5]. 

The great majority of mammals synthesize vitamin C in their bodies, but primates and the guinea pig cannot. Therefore, the guinea pig is a useful animal model on which to study vitamin C deficiency. Bacteria were often found in histological sections of scorbutic guinea pigs, so much so that some early authors assumed that scurvy might be an infectious disease. However, Hess (1920) concluded that such results merely showed that the tissues of scorbutic animals frequently harbor bacteria, and “there is no doubt that the invasion of the blood-stream does occur readily in the course of scurvy, but this takes place generally after the disease has developed and must be regarded as a secondary phenomenon and therefore unessential from an etiological standpoint. Indeed one of the striking and important symptoms of scurvy is the marked susceptibility to infection” [3]. When summarizing autopsy findings of experimental scurvy in the guinea pig, Hess also noted that “Pneumonia is met with very frequently and constitutes a common terminal infection”.

Vitamin C was considered as an explanation for scurvy, which was regarded as a disease of the connective tissues, since many of the symptoms such as poor wound healing implied crucial effects on the connective tissues. Therefore, the mainstream view in medicine regarded vitamin C as a vitamin that safeguards the integrity of connective tissues [6]. The implications of the earlier research by Hess and others were superseded. This historical background might explain the current lack of interest in the effects of vitamin C on infections, even though firm evidence that vitamin C influences infections has been available for decades.

Early literature on vitamin C and infections was reviewed by Clausen (1934), Robertson (1934), and Perla and Marmorston (1937) [5,7,8]. Those reviews are thorough descriptions of the large number of early studies on the topic of this review. Scanned versions of those reviews and English translations of many non-English papers cited in this review are available at the home page of this author [9]. The book on scurvy by Hess (1920) is available in a digitized format [3].

## 2. Biology Relevant to the Effects of Vitamin C on Infections

Evidence-based medicine (EBM) emphasizes that in the evaluation of treatments researchers should focus primarily on clinically relevant outcomes, and little weight should be put on biological explanations. Therefore, this review focuses on infections and not on the immune system. Immune system effects are surrogates for clinical effects and there are numerous cases when surrogates had poor correlations with clinically relevant outcomes [10]. Nevertheless, biology provides a useful background when we consider the plausibility of vitamin C to influence infections.

### 2.1. Dose–Concentration Relationship

The vitamin C level in plasma of people in good health becomes saturated at about 70 µmol/L when the intake is about 0.2 g/day [11]. On the other hand, when vitamin C intake is below 0.1 g/day, there is a steep relationship between plasma vitamin C level and the dose of the vitamin. Clinical scurvy may appear when the plasma concentration falls below 11 µmol/L, which corresponds to an intake of less than 0.01 g/day [12,13,14]. Thus, when healthy people have a dietary intake of about 0.2 g/day of vitamin C, there is usually no reason to expect a response to vitamin C supplementation. This does not apply universally because certain studies have shown the benefits of supplementation, even though the baseline intake was as high as 0.5 g/day (see below). If the initial vitamin C intake is lower than about 0.1 g/day, effects of vitamin C supplementation may be expected on the basis of the dose–concentration curve. Nevertheless, this argument does not apply to patients with infections since their vitamin C metabolism is altered and they have decreased vitamin C levels (see below).

### 2.2. Infections Increase Oxidative Stress

Vitamin C is an antioxidant. Therefore, any effects of vitamin C may be most prominent under conditions when oxidative stress is elevated. Many infections lead to the activation of phagocytes, which release oxidizing agents referred to as reactive oxygen species (ROS). These play a role in the processes that lead to the deactivation of viruses and the killing of bacteria [15]. However, many of the ROS appear to be harmful to the host cells, and in some cases they seem to play a role in the pathogenesis of infections [16,17]. Vitamin C is an efficient water-soluble antioxidant and may protect host cells against the actions of ROS released by phagocytes. Phagocytes have a specific transport system by which the oxidized form of vitamin C (dehydroascorbic acid) is imported into the cell where it is converted into the reduced form of vitamin C [18,19]. 

Influenza A infection in mice resulted in a decrease in vitamin C concentration in bronchoalveolar lavage fluid, which was concomitant with an increase in dehydroascorbic acid, the oxidized form of vitamin C [20], and in vitamin C deficiency influenza led to greater lung pathology [21]. Respiratory syncytial virus decreased the expression of antioxidant enzymes thereby increasing oxidative damage [22]. Bacterial toxins have also led to the loss of vitamin C from many tissues in animal studies [1] (p. 6). 

Increased ROS production during the immune response to pathogens can explain the decrease in vitamin C levels seen in several infections. There is evidence that plasma, leukocyte and urinary vitamin C levels decrease in the common cold and in other infections [1,23]. Hume and Weyers (1973) reported that vitamin C levels in leukocytes halved when subjects contracted a cold and returned to the original level one week after recovery [24]. Vitamin C levels are also decreased by pneumonia [25,26,27,28]. 

Decreases in vitamin C levels during various infections imply that vitamin C administration might have a treatment effect on many patients with infections. There is no reason to assume that the saturation of plasma or leukocyte vitamin C levels during infections is reached by the 0.2 g/day intake of vitamin C that applies to healthy people (see above). In particular, Hume and Weyers (1973) showed that supplementation at the level of 0.2 g/day was insufficient to normalize leukocyte vitamin C levels in common cold patients, but when 6 g/day of vitamin C was administered, the decline in leukocyte vitamin C induced by the common cold was essentially abolished [24].

### 2.3. Vigorous Physical Activity Increases Oxidative Stress

Heavy physical stress leads to the elevation of oxidative stress [29]. Therefore, responses to vitamin C might be observed when people are particularly active physically. Electron spin resonance studies have shown that vitamin C administration decreased the levels of free radicals generated during exercise [30] and vitamin C administration attenuated the increases in oxidative stress markers caused by exercise [31]. Therefore, vitamin C supplementation might have beneficial effects on people who are under physical stress. In such cases there is no reason to assume that 0.2 g/day of vitamin C might lead to maximal effects of the vitamin. Direct evidence of benefits of vitamin C supplementation to physically active people was found in three randomized trials in which 0.5 to 2 g/day of vitamin C prevented exercise-induced bronchoconstriction [32,33].

### 2.4. Vitamin C May Protect against Stress Caused by Cold and Hot Environments

Studies in animals and humans have indicated that vitamin C may protect against stress caused by cold and hot environments [34,35,36,37]. Some common cold studies with positive results investigated physically active participants in cold environments and other studies investigated marathon runners in South Africa (see below). Therefore, the effects of vitamin C in the protection against cold or heat stress might also be relevant when explaining the benefits in those studies.

### 2.5. Marginally Low Vitamin C Status Might Lead to Benefits of Supplementation

It seems evident that any effects of vitamin C supplementation may be more prominent when the baseline vitamin C level is particularly low. As noted above, a profound vitamin C deficiency was associated with pneumonia in the early literature. It seems plausible that less severe vitamin C deficiency, which may be called “marginal vitamin C deficiency”, can also be associated with increased risk and severity of infections, although the effects may be less pronounced than those caused by scurvy.

Low vitamin C levels are not just of historical relevance. Cases of scurvy in hospitals have been described in several recent case reports [38,39]. One survey estimated that about 10% of hospitalized elderly patients had scurvy [40]. Surveys have also shown that plasma vitamin C levels below 11 µmol/L were found for 14% of males and 10% of females in the USA, 19% of males and 13% of females in India, 40% of elderly people living in institutions in the UK, 23% of children and 39% of women in Mexico, and 79%–93% of men in Western Russia. Moreover, 45% of a cohort of pregnant women in rural India had plasma vitamin C levels below 4 µmol/L and the mean plasma vitamin C level fell to 10 µmol/L in a cohort of pregnant or lactating women in Gambian villages in the rainy season [41]. 

The mean vitamin C intake in adults in the USA has been about 0.10 g/day, but 10% of the population has had intake levels of less than 0.04 g/day [14]. Thus, if low intake levels of vitamin C have adverse effects on the incidence and severity of infections, this may be important also in population groups in western countries, and not just in developing countries.

### 2.6. Vitamin C Has Effects on the Immune System 

Vitamin C levels in white blood cells are tens of times higher than in plasma, which may indicate functional roles of the vitamin in these immune system cells. Vitamin C has been shown to affect the functions of phagocytes, production of interferon, replication of viruses, and maturation of T-lymphocytes, etc. in laboratory studies [1,23,42,43,44]. Some of the effects of vitamin C on the immune system may be non-specific and in some cases other antioxidants had similar effects. 

### 2.7. The Diverse Biochemical, Physiological, and Psychological Effects of Vitamin C

Biochemistry textbooks usually mention the role of vitamin C in collagen hydroxylation. However, the survival time of vitamin C deficient guinea pigs was extended by carnitine [45] and by glutathione [46], which indicates that scurvy is not solely explained by defects in collagen hydroxylation, and it is not clear whether hydroxylation is important at all in explaining scurvy [6]. Vitamin C participates in the enzymatic synthesis of dopamine, carnitine, a number of neuroendocrine peptides, etc. [6,47,48,49,50]. Vitamin C is also a powerful antioxidant, as mentioned above. 

Experimentally induced vitamin C deficiency leads to depression and fatigue [11,51]. Recently, vitamin C was reported to improve the mood of acutely hospitalized patients [52,53]. Such effects cannot be explained by collagen metabolism, and vitamin C effects on the immune system are not plausible explanations either. Instead, the effects of vitamin C on the neuroendocrine system or carnitine metabolism might explain such effects. Thus, if vitamin C has beneficial effects on patients with infections, that does not unambiguously indicate that these effects are mediated by the immune system per se. 

### 2.8. The Effects of Antioxidants against Infections May Be Heterogeneous

It is quite a common assumption that the effects of vitamins are uniform. Thus, if there is benefit, it is often assumed that the same benefit applies to all people. However, it seems much more likely that the effects of vitamins, including vitamin C, vary between people depending on biology and their lifestyle. Thus, it is possible that there are benefits (or harms) restricted to special conditions or to particular people. In the case of vitamin E, there is very strong evidence for the heterogeneity in its effects on pneumonia [54,55] and on the common cold [56]. Although the factors modifying the effects of vitamin E cannot be extrapolated to vitamin C, it seems probable that there is comparable heterogeneity in the effects of vitamin C.

## 3. Infections in Animals

Early research showed that severe deficiency of vitamin C increased the incidence and severity of infections in guinea pigs. Hemilä (2006) carried out a systematic search of animal studies on vitamin C and infections and analyzed their findings [1], which are summarized in Table 1, Table 2 and Table 3 and discussed below. 

### 3.1. Studies with Diets Containing Vitamin C 

Many early studies with guinea pigs did not examine the effect of pure vitamin C. Instead, “vitamin-C-deficient groups” were fed diets that contained only small amounts of vitamin C, whereas the “vitamin C group” was administered oranges or other foods that contained high levels of vitamin C. The findings of studies on guinea pigs with tuberculosis and other bacterial infections are shown in Table 1.

Assuming that vitamin C containing foods do not influence infections, by pure chance only, one positive result at the level of *p* < 0.01 would be expected for a group of 100 studies. However, 20 of the reported 28 studies found significant benefits from feeding diets rich in vitamin C (Table 1). Although these findings are consistent with the notion that low vitamin C intake may increase the susceptibility to and the severity of infections, other substances in fruit and vegetables might also contribute to this effect, thus confounding the differences between the study groups. 

As one example of Table 1 studies, McConkey (1936) reported that the administration of tuberculous sputum to 16 guinea pigs that were vitamin C deficient led to intestinal tuberculosis to 15 of them, but none of the five guinea pigs that were administered tomato juice as a source of vitamin C suffered from intestinal tuberculosis [57] (pp. 507–508).

### 3.2. Studies with Pure Vitamin C

Table 2 summarizes the animal studies in which pure vitamin C was administered to the “vitamin C” group. Overall, 148 animal studies had been published by 2005. 

Out of the 148 studies, over half found a significant benefit, *p* < 0.01, for at least one infectious disease outcome. Furthermore, over a third of the studies found a benefit at the level of *p* < 0.001 [1]. Of the 100 studies with mammals, 58 found a significant benefit, *p* < 0.01, from vitamin C on some infectious disease outcome.

A benefit of vitamin C against infections was found in all animal groups. Although rats and mice synthesize vitamin C in their bodies, half or more of the studies with these species found significant benefits of additional vitamin C. This implies that rats and mice do not necessarily synthesize sufficient amounts of vitamin C to reach optimal levels that prevent or curtail infections. In addition to mammals, vitamin C protected against infections in several studies with birds and fishes. 

Vitamin C was found to be beneficial against various groups of infectious agents including bacteria, viruses, *Candida albicans*, and protozoa (Table 2). Over half (*n* = 97) of all the studies evaluated the effect of vitamin C on bacterial infections or bacterial toxins, and 55 out of those studies found significant benefits of vitamin C (*p* < 0.01). Studies in which animals were administered diphtheria toxin, tetanus toxin, or endotoxin are also relevant, because these toxins are essential components in the pathogenesis of the bacterial infections. Over half of the studies on viruses, *Candida albicans* and protozoa also reported significant benefits (*p* < 0.01). 

Table 3 shows the distribution of infections in studies that reported decreases in mortality caused by infections (*p* < 0.001). It is apparent that vitamin C reduced mortality in all etiological groups. 

As one example of the studies in Table 2 and Table 3, Dey (1966) reported that five rats administered twice the minimal lethal dose of tetanus toxin all died, whereas 25 rats administered vitamin C either before or after the same dose of toxin all lived [58].

In addition to the animal studies yielding quantitative data on the effect of vitamin C on infections in Table 1, Table 2 and Table 3, a few studies reported interesting findings of vitamin C effects against infections in studies without control groups [1] (p. 9). For example, two case-series suggested therapeutic benefit of vitamin C on dogs afflicted by the canine distemper virus. Belfield (1967) described a series of 10 dogs that appeared to benefit from 1–2 g/day of intravenous vitamin C over three days [59]. Leveque (1969) noted that usually only 5%–10% of dogs recovered from canine distemper with signs of central nervous system (CNS) disturbance. He became interested in Belfield’s report and in a series of 16 dogs showing CNS disturbance that were treated with vitamin C, the proportion of dogs that recovered was 44% (95% CI: 20%–70%; based on 7/16) [60].

### 3.3. Implications of the Animal Studies

Many of the studies on vitamin C and infections summarized in Table 2 are old. However, it is unlikely that administering a specified dose of pure vitamin C and evaluating clinical outcomes of infections, such as mortality, will have changed meaningfully since those early days. Furthermore, 60 studies were published in the 1990s or later, and half of these later reports also found significant benefits of vitamin C on at least one infectious disease outcome.

The studies on guinea pigs are most interesting since that species is dependent on dietary vitamin C as are humans. Infections in guinea pigs against which vitamin C was significantly beneficial included *Mycobacterium tuberculosis*, *β-*hemolytic streptococci, *Fusobacterium necrophorum*, diphtheria toxin, *Entamoeba histolytica*, *Trypanosoma brucei*, and *Candica albicans* [1].

Some of the 148 studies in Table 2 were small and did not have sufficient statistical power to test whether vitamin C and control groups might differ. However, this problem cannot explain the large number of reported significant benefits. In contrast, inclusion of studies with a low statistical power biases the findings towards the opposite direction, leading to false negative findings. 

Mortality and severity of infections in animals are definitive outcomes. In this respect, the animal studies with actual infections are much more relevant to humans than studies on laboratory determinations of the human immune system.

Given the universal nature of the effect of vitamin C against infections in diverse animal species as seen in Table 2, it seems obvious that vitamin C also has influences on infections in humans. It seems unlikely that human beings qualitatively differ from all of the animal species that have been used in the experiments listed in Table 2. Nevertheless, it is not clear to what degree the animal studies can be extrapolated to human subjects. 

The fundamental question in human beings is not whether vitamin C affects the susceptibility to and severity of infections. Instead, the relevant questions are the following: What are the population groups who might benefit from higher vitamin C intakes? What is the dose-dependency relation between intake and the effects on infections? How does the optimal level of intake differ between healthy people and patients with infections?

## 4. The Common Cold

The term “the common cold” does not refer to any precisely defined disease, yet the set of symptoms that is called “the common cold” is personally familiar to practically everybody [61]. Typically the symptoms consist of nasal discharge, sore throat, cough, with or without fever. Young children typically have half a dozen colds per year, and the incidence decreases with age so that elderly people have colds about once per year [62]. The common cold is the leading cause of acute morbidity and of visits to a physician in high-income countries, and a major cause of absenteeism from work and school. The economic burden of the common cold is comparable to that of hypertension or stroke [63].

The most relevant definition of the common cold is based on the symptoms; thus the “common cold” does not always entail a viral etiology. Although the majority of common cold episodes are caused by respiratory viruses, similar symptoms are also caused by certain bacterial infections and by some non-infectious causes such as allergic and mechanical irritation. The cough and sore throat after running a marathon does not necessarily imply a viral etiology, although some researchers have assumed so. It is still reasonable to use the term the “common cold” in such a context on the grounds of the symptom-based definition.

### 4.1. Vitamin C and the Common Cold

Interest in the effects of vitamin C on the common cold originated soon after purified vitamin C became available. The first controlled trials on vitamin C were carried out as early as the 1940s. For example, in the 1950s, a British study examined the clinical effects of vitamin C deprivation, and reported that “the geometric mean duration of colds was 6.4 days in vitamin C-deprived subjects and 3.3 days in non-deprived subjects”, and the authors concluded that the absence of vitamin C tended to cause colds to last longer [12].

**Figure 1:**
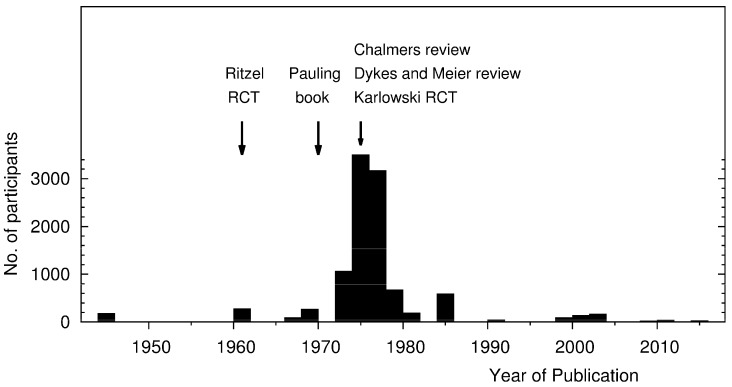
The numbers of participants in the placebo-controlled trials for which ≥1 g/day of vitamin C was administered. The numbers of participants in studies published over two consecutive years are combined and plotted for the first of the two years. This figure is based on data collected by Hemilä and Chalker (2013) [68,69]. See Supplementary file 1 of this review for the list of the studies. RCT, randomized controlled trial.

Figure 1 shows the number of participants in placebo-controlled studies in which ≥1 g/day of vitamin C was administered. It also illustrates the main time points of the history of vitamin C and the common cold.

In 1970, Linus Pauling, a Nobel laureate in chemistry and also a Nobel Peace Prize winner, wrote a book on vitamin C and the common cold [64]. He also published two meta-analyses, which were among the earliest meta-analyses in medicine [65,66]. Pauling identified four placebo-controlled studies from which he calculated that there was strong evidence that vitamin C decreased the “integrated morbidity” of colds (*p* = 0.00002 [65]). By integrated morbidity, Pauling meant the total burden of the common cold: the combination of the incidence and duration of colds. In his analysis, Pauling put the greatest weight on the study by Ritzel (1961), which was a randomized controlled trial (RCT) with double-blinded placebo control and the subjects were schoolchildren in a skiing camp in the Swiss Alps [67]. Ritzel’s study was methodologically the best of the four and used the highest dose of vitamin C, 1 g/day, and therefore Pauling concluded that gram doses of vitamin C would be beneficial against colds [64,65,66].

The activity of Pauling, in turn, led to a great upsurge in interest in vitamin C among lay people and also in academic circles in the early 1970s. From 1972 to 1979, in that eight-year period, 29 placebo-controlled studies were published, which amounted to a total of 8409 participants (Figure 1) [68,69]. Thus, the mean number of participants per study was 290.

In the interval from 1972 to 1975, five placebo-controlled trials were published that used ≥2 g/day of vitamin C. Those five studies were published after Pauling’s book and therefore they formally tested Pauling’s hypothesis. A meta-analysis by Hemilä (1996) showed that there was very strong evidence from the five studies that colds were shorter or less severe in the vitamin C groups (*p* = 10^−5^), and therefore those studies corroborated Pauling’s hypothesis that vitamin C was indeed effective against colds [70]. 

After the mid-1970s, however, interest in the topic plummeted so much so that during the 30-year period from 1985 to 2014, only 11 placebo-controlled trials comprising just 538 participants in total were published, with a mean of 49 participants per study (Figure 1). Thus, the number of studies published after 1985 is much lower than during the 1970s. In addition, the few recent studies are much smaller than the trials published in the 1970s. Therefore, the great majority of the data on vitamin C and the common cold that are currently available originated within the decade after the publication of Pauling’s book.

This sudden lack of interest after the middle of the 1970s can be explained by three papers published in the same year by Chalmers (1975), Karlowski et al. (1975), and Dykes and Meier (1975) [71,72,73] (Figure 1). Few trials were started after 1975, which indicates the great impact of these three papers. First, the findings of the placebo controlled studies will be summarized, and then difficulties in the interpretation of common cold studies will be considered, and finally problems in the three papers that were published in 1975 will be discussed. 

### 4.2. Vitamin C Does Not Decrease the Average Incidence of Colds in the General Community

Table 4 summarizes the findings of the studies on vitamin C and the common cold in the Cochrane review by Hemilä and Chalker (2013) [68,69]. Regularly administered vitamin C has not decreased the average number of colds among the general population (Table 4). Another meta-analysis combined the findings of the six largest trials that had used ≥1 g/day of vitamin C and calculated that there was no difference in the vitamin and placebo groups with RR = 0.99 (95% CI 0.93, 1.04) [74,75].

Thus, there is no justification for “ordinary people” to take vitamin C regularly in order to prevent colds. However, this conclusion does not mean that regular vitamin C supplementation is ineffective for all people. There is strong evidence that vitamin C decreases the incidence of colds under special conditions and/or among certain population groups.

### 4.3. Vitamin C May Decrease Common Cold Incidence in Special Conditions

Vitamin C halved the incidence of colds in five RCTs during which the participants were under heavy short-term physical activity (Table 4) [68,76]. Three of the studies used marathon runners in South Africa as subjects, whereas one study used Canadian military personnel on winter exercise, and the fifth study was on schoolchildren in a skiing camp in the Swiss Alps, i.e., the Ritzel (1961) trial [67]. Thus, three studies were conducted under conditions of a hot environment and profound physical stress and the other two were carried out under cold environments and physical stress (see Section 2.4).

Another group in which vitamin C has prevented colds is British men [74,75,77]. Four trials found that vitamin C decreased the incidence of colds by 30%, and in another set of four trials, the proportion of men who had recurrent common cold infections during the study decreased by a mean of 46%. All these studies were carried out in the 1970s or earlier, and according to surveys, the intake of vitamin C in the United Kingdom was low when the studies were carried out, 0.03 to 0.06 g/day, and three of the U.K. trials specifically estimated that the dietary vitamin C intake was between 0.015 to 0.05 g/day [74]. In particular, Baird (1979) administered only 0.08 g/day of vitamin C yet they observed 37% lower incidence of colds in the vitamin C group, indicating that it was the “marginal deficiency” and not a high dose that explained the benefit [77,78]. 

In addition, the levels of vitamin C are usually lower in men than in women, which may explain the benefit for British males, in comparison to no apparent effect in British females. Evidently, the dietary vitamin C intake in the United Kingdom has increased since the 1970s, and therefore these studies do not indicate that vitamin C supplementation would necessarily influence colds in ordinary British men nowadays. However, if low dietary vitamin C intake increases the risk of respiratory infections, then that may be currently relevant in other contexts, since there are still many population groups that have low intakes of vitamin C. A recent small study in the USA by Johnston (2014) was restricted to 28 males with marginally low vitamin C levels, mean 30 µmol/L, and found a decrease in common cold incidence, RR = 0.55 (95% CI: 0.33–0.94; *p* = 0.04) [79], which may also be explained by the low vitamin C levels.

### 4.4. Vitamin C Might Protect against the Common Cold in a Restricted Subgroup of the General Community

Although vitamin C has not influenced the average common cold incidence in the general community trials (Table 4), some of them found that there was a subgroup of people who had obtained benefits from vitamin C. In a Canadian trial, Anderson (1972) [80] reported that in the vitamin C group there were 10 percentage points more participants with no “days confined to house” because of colds (57% vs. 47%; *p* = 0.01, [1] (p. 44)). Thus, one in 10 benefited from vitamin C in this outcome. In a trial with Navajo schoolchildren, Coulehan (1974) [81] found that in the vitamin C group there were 16 percentage points more children who were “never ill on active surveillance by a medically trained clerk or the school nurse” (44% vs. 29%; *p* < 0.001; [1] (p. 44)). A more recent study in the UK by van Straten (2002) reported that vitamin C decreased the number of participants who had recurrent colds by 17 percentage points [82] (19% vs. 2%; *p* < 0.001, [1] (p. 47)). Thus, the statistical evidence of benefit for a restricted subgroup in these three trials is strong.

### 4.5. Vitamin C Shortens and Alleviates the Common Cold

The effect of vitamin C on the duration and severity of the common cold has been studied in regular supplementation trials and in therapeutic trials. Regular supplementation means that vitamin C was administered each day over the whole study period, and the outcome is the duration and severity of colds that occurred during the study. Therapeutic vitamin C trial means that vitamin C administration was started only after the first common cold symptoms had occurred and the duration of colds were then recorded. 

In regular supplementation studies, ≥0.2 g/day of vitamin C decreased the duration of colds by 9% (Table 4). When the dosage was ≥1 g/day of vitamin C, the mean duration of colds was shortened by 8% in adults and by 18% in children. Vitamin C also significantly alleviated the severity of the colds. 

**Figure 2:**
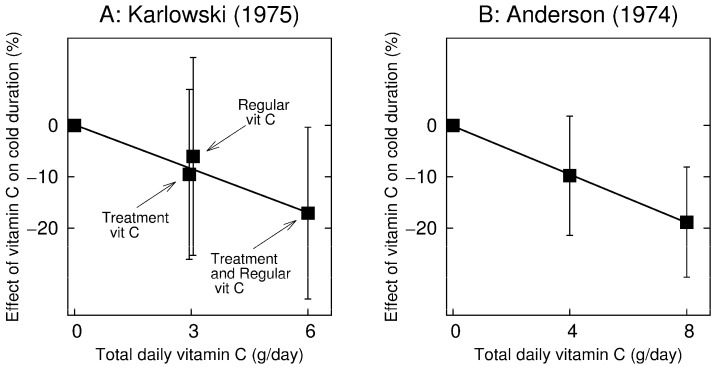
(**A**) Dose–response relationship in the Karlowski (1975) trial. The placebo arm is located at 0 g/day, the 3 g/day regular vitamin C and the 3 g/day treatment vitamin C arms are in the middle and the regular + treatment arm is at 6 g/day [72]. The 95% CIs are shown for the comparison against the placebo arm. With inverse-variance weighing, test for trend in a linear model gives *p*(2-tail) = 0.018. The addition of the linear vitamin C effect to the statistical model containing a uniform vitamin C effect improved the regression model by *p* = 0.002. Previously, analysis of variance for trend calculated *p* = 0.040 for the linear trend [83]; (**B**) Dose–response relationship in the Anderson (1974) trial. The placebo arm #4 is located at 0 g/day, vitamin C treatment arm #7 at 4 g/day and vitamin C treatment arm #8 at 8 g/day [84]. In the Anderson (1974) trial, vitamin C was administered only on the first day of the common cold. The 95% CIs are shown for the comparison against the placebo arm. With inverse-variance weighing, test for trend in a linear model gives *p*(2-tail) = 0.013. See Supplementary file 2 for the calculation of the trend for both studies.

Therapeutic studies have hitherto not shown consistent benefit from vitamin C. However, therapeutic trials are more complex to conduct and interpret than regular supplementation trials. If the timing of the initiation of supplementation or the duration of supplementation influences the extent of the benefit, false negative findings may result from inappropriate study protocols. For example, four therapeutic studies used only 2–3 days of 2–4 g/day vitamin C supplementation, whereas the mean duration of colds in these studies was about a week. None of these studies detected any benefit from vitamin C [68,83]. On the other hand, Anderson (1974) [84] found that 8 g/day on the first day only reduced the duration of colds significantly (Figure 2). In addition, in a five-day therapeutic trial, Anderson (1975) [85] reported a 25% reduction in “days spent indoors per subject” because of illness (*p* = 0.048) in the vitamin C group (1 to 1.5 g/day) [1] (p. 48). Finally, none of the therapeutic studies investigated children, although the effect of regular vitamin C has been greater in children (Table 4). Thus, although the regular supplementation trials unambiguously show that vitamin C shortens and alleviates the common cold, there is no consistent evidence that therapeutic supplementation is effective.

### 4.6. Possible Differences in the Effects of Vitamin C between Subgroups

The regular supplementation study by Anderson (1972) is one of the largest that has been carried out [80]. They found that the proportion of participants who were not confined to the house decreased by 10 percentage points in the vitamin C group. In addition, they found that per episode the days confined to the house was 21% shorter in the vitamin C group. Together these combine to a 30% reduction in the days confined to the house per person (*p* = 0.001). Such a large effect gives statistical power for subgroup comparisons.

Anderson (1972) reported that vitamin C decreased total days confined to house by 46% in participants who had contact with young children, but just by 17% in participants who did not have contact with young children (Table 5). Anderson (1972) also reported that vitamin C decreased total days confined to house by 43% in participants who usually had two or more colds per winter, but just by 13% in participants who usually had zero to one cold per winter (Table 5).

In a study with adolescent competitive swimmers, Constantini (2011) found a significant difference between males and females in the effect of vitamin C, whereby the vitamin halved the duration and severity of colds in males but had no effect on females [86]. In a study with British students, Baird (1979) also found a significant difference between males and females, but the outcome was the incidence of colds (Table 5).

Carr (1981) found that vitamin C had a beneficial effect on the duration of colds for twins living separately, but not for twins living together [87]. This subgroup difference might be explained by swapping of tablets by twins living together, which was not possible for twins living separately.

The significant within-trial differences in the effect of vitamin C on the common cold indicate that there is no universal effect of vitamin C valid over the whole population. Instead, the size of the vitamin C effect seems to depend on various characteristics of people (see Section 2.8).

### 4.7. Dose Dependency of Vitamin C Supplementation Effect

An earlier meta-analysis of dose-dependency calculated that on average 1 g/day of vitamin C shortened the duration of colds in adults on average by 6% and in children by 17%; and ≥2 g/day vitamin C shortened the duration of colds in adults by 21% and in children by 26% [83]. Thus, higher doses were associated with greater effects. In addition, children weigh less than adults and the greater effects in children may be explained by a greater dose per weight. Nevertheless, such a comparison suffers from numerous simultaneous differences between the trials. The most valid examination of dose–response is within a single study so that the virus distribution is similar in each trial arm and the outcome definition is identical.

Coulehan (1974) [81] administered 1 g/day to children and observed a 12% reduction in common cold duration, and in parallel they administered 2 g/day to other children and observed a 29% reduction in cold duration. Although the point estimates suggest a dose–response, the study was small and the 95% CIs overlap widely [68,83].

In a 2 × 2 design, Karlowski (1975) [72] randomized participants to 3 g/day regular vitamin C and to 3 g/day vitamin C treatment for five days when the participant caught a cold. Thus, one study arm was administered placebo, the second was administered regular vitamin C, the third therapeutic, and the fourth arm was administered regular + therapeutic vitamin C (i.e., 6 g/day). The four arms of the Karlowski trial are shown in Figure 2A. The 95% CIs show the comparisons with the placebo group. The test for trend for a linear regression model gives *p* = 0.018.

Anderson (1974) [84] randomized participants to a placebo and two vitamin C treatment arms which were administered vitamin C only on the first day of the cold. One treatment arm (arm #7) was given 4 g/day of, and another (arm #8) was given 8 g/day. These arms are compared with the placebo arm #4 in Figure 2B. The 95% CIs show the comparisons with the placebo group. The test for trend in a linear regression model gives *p* = 0.013.

Finally, some case reports have proposed that vitamin C doses should be over 15 g/day for the best treatment of colds [88,89]. Thus, it is possible that the doses used in most of the therapeutic studies, up to just 6–8 g/day, have not been sufficiently high to properly test the effects of vitamin C that might be achievable.

### 4.8. Vitamin C and Complications of the Common Cold

Given the strong evidence that regularly administered vitamin C shortens and alleviates common cold symptoms, it seems plausible that vitamin C might also alleviate complications of the common cold. One frequent complication is the exacerbation of asthma [90]. 

A systematic review identified three studies that provided information on the potential pulmonary effects of vitamin C in sufferers of common cold–induced asthma [91]. A trial conducted in Nigeria studied asthmatic patients whose asthma exacerbations resulted from respiratory infections. A vitamin C dose of 1 g/day decreased the occurrence of severe and moderate asthma attacks by 89% [92]. Another study on patients who had infection-related asthma reported that 5 g/day vitamin C decreased the prevalence of bronchial hypersensitivity to histamine by 52 percentage points [93]. A third study found that the administration of a single dose of 1 g vitamin C to non-asthmatic common cold patients decreased bronchial sensitivity in a histamine challenge test [94].

It has also been proposed that vitamin C might prevent sinusitis and otitis media [95,96], but to our knowledge there are no data from controlled studies.

A further complication of viral respiratory infections is pneumonia; this is discussed in the section on pneumonia.

## 5. Problems in the Interpretation: Non-Comparability of the Vitamin C and Common Cold Trials

### 5.1. Vitamin C Doses in Vitamin C and Control Groups

One great problem in the interpretation of vitamin C trials arises from the fundamental difference between vitamin C and ordinary drugs such as antibiotics. In a trial of an ordinary drug, the control group is not given the drug, which simplifies the interpretation of the findings. In contrast, it is impossible to select control subjects who have zero vitamin C intake and no vitamin C in their system. Thus, all vitamin C trials de facto compare two different vitamin C levels. The lower dose is obtained from the diet, and it has varied considerably among the controlled studies. In addition, the vitamin C supplement doses given to the vitamin C groups have also varied extensively. Finally, the placebo group in some trials was also given extra vitamin C, which further confuses the comparisons. Therefore, the comparison of different vitamin C studies and the generalization of their findings is complicated. As an illustration of these problems, Table 6 shows examples of the variations in vitamin C doses that were used in the common cold trials. 

There are 10- to 30-fold differences in the vitamin C intake in the diet of the control groups of the Baird (1979) [78], the Glazebrook (1942) [97], and the Sabiston (1974) [98] trials compared with the Peters (1993) [99] trial, yet all of them are labeled “control groups” of vitamin C trials (Table 6). Evidently, we should not expect similar effects of supplemental vitamin C in such dissimilar studies. Usually the dietary intake of vitamin C is not estimated and therefore cannot be taken into account when comparing studies. 

Vitamin C was administered to the placebo group in some studies. For example, Carr (1981) [87] administered 0.07 g/day and some other studies administered 0.01 to 0.05 g/day to the control subjects. This was done to refute the notion that any possible effects of high doses were due to the treatment of marginal deficiencies. Such reasoning does not seem sound, since there are population groups for which ordinary dietary vitamin C intake is particularly low and it would be important to know whether vitamin C supplementation might be beneficial for them. Thus, marginal vitamin C deficiency is also an important issue. The administration of vitamin C to the control group biases the possible effects of vitamin C supplementation downwards. 

Finally, there are up to a 240-fold difference between the lowest and highest vitamin C supplementary dose used in the common cold trials, yet the dosage is often ignored. For example, in his influential review (see Figure 1), Chalmers (1975) [71] presented data from the following studies in the same table: Karlowski (1975) study administered up to 6 g/day of vitamin C to their subjects [72], whereas the Cowan (1942) study administered only 0.025 g/day as the lowest dose [100]. Chalmers (1975) did not list the vitamin C dosages in his table and therefore his readers were unable to consider whether the comparison of such different studies was reasonable or not. Still, Chalmers’ review has been widely cited as evidence that vitamin C is not effective against colds [1] (pp. 36–38).

Finally, combinations of the above variations lead to paradoxes. The “vitamin C group” of the Baird (1979) study received about 0.05 g/day of vitamin C from food and 0.08 g/day from supplements, which amounted to 0.13 g/day of total vitamin C [78]. In contrast, the “placebo group” in the study by Peters (1994) received about four times as much, 0.5 g/day, of vitamin C from their usual diet [99]. Furthermore, Baird (1979) administered 0.08 g/day of vitamin C to their vitamin C group [78], whereas Carr (1981) administered 0.07 g/day vitamin C to their placebo group [87]. Thus, the dosages of vitamin C were essentially the same, but the groups were on the opposite sides in the evaluation of vitamin C effects.

High dietary vitamin C intake, and vitamin C supplementation of the placebo group, cannot lead to false positive findings about the efficacy of vitamin C against colds. In contrast, they can lead to false negative findings or estimates biased towards the null effect. 

### 5.2. Non-Compliance of Participants

Carr (1981) studied twins, some of whom lived together, whereas others lived apart [87]. Vitamin C had a significant effect on the duration and severity of colds in twins living apart, but no effect in twins living together (Table 5). Furthermore, the duration of colds among twins living together (5.4 days in vitamin C and placebo groups) was in the middle of the duration of colds among the vitamin C group (4.9 days) and placebo group (7.5 days) of twins living apart. An evident explanation for such a difference between twins living together and twins living apart, is that twins who lived together exchanged their tablets to some extent, whereas the twins who lived apart could not do so. Two studies on children found an increase in vitamin C levels in the plasma of boys and in the urine of boys of the placebo (sic) groups [81,101], which indicates tablet swapping among the children on vitamin C and placebo. Thus, non-compliance may have confounded the results and the true effects of vitamin C might be greater than those reported.

### 5.3. Implications of the Common Cold Studies

Given the great variations in the vitamin C dosage levels in the vitamin C and control groups, and the apparent problem of non-compliance in some studies, it is obvious that the comparison of different “vitamin C trials” can be complicated. The generalization of the findings of any particular trial is limited irrespective of its methodological quality and statistical power. However, the large variations in vitamin C levels in the vitamin C and control groups, and the non-compliance in some studies, both predispose against a false positive differences between the study groups. In contrast, they make it more difficult to detect true differences, and therefore the findings on common cold duration and severity shown in Table 4 may be biased downwards and might camouflage even stronger true effects.

## 6. Evaporation of Interest in Vitamin C and the Common Cold after 1975

Given the strong evidence from studies published before 1970 that vitamin C has beneficial effects against the common cold [65], and from the ≥2 g/day vitamin C studies published between 1972 and 1975 [70], it is puzzling that the interest in vitamin C and the common cold collapsed after 1975 so that few small trials on vitamin C and the common cold have been conducted thereafter (Figure 1).

This sudden loss of interest can be explained by the publication of the three highly important papers in 1975 (Figure 1). These papers are particularly influential because of their authors and the publication forums. Two of the papers were published in *JAMA* [72,73], and the third paper was published in the *American Journal of Medicine* [71]. Both of these journals are highly influential medical journals with extensive circulations. Two of the papers were authored by Thomas Chalmers [71,72], who was a highly respected and influential pioneer of RCTs [1,102,103], and the third paper was authored by Paul Meier [73], who was a highly influential statistician, e.g., one of the authors of the widely used Kaplan–Meier method [1,104,105].

Karlowski, Chalmers, et al. (1975) [72] published the results of a RCT in *JAMA*, in which 6 g/day of vitamin C significantly shortened the duration of colds (Figure 2A). However, these authors claimed that the observed benefit was not caused by the physiological effects of vitamin C, but by the placebo effect. However, the “placebo-effect explanation” was shown afterwards to be erroneous. For example, Karlowski et al. had excluded 42% of common cold episodes from the subgroup analysis that was the basis for their conclusion, without giving any explanation of why so many participants were excluded. The numerous problems of the placebo explanation are detailed in a critique by Hemilä [1,106,107]. Chalmers wrote a response [108], but did not answer the specific issues raised [109]. 

In the same year (1975), Chalmers published a review of the vitamin C and common cold studies. He pooled the results of seven studies and calculated that vitamin C would shorten colds only by 0.11 (SE 0.24) days [71]. Such a small difference has no clinical importance and the SE indicates that it is simply explained by random variation. However, there were errors in the extraction of data, studies that used very low doses of vitamin C (down to 0.025 g/day) were included, and there were errors in the calculations [1,110]. Pauling had proposed that vitamin C doses should be ≥1 g/day. When Hemilä and Herman (1995) included only those studies that had used ≥1 g/day of vitamin C and extracted data correctly, they calculated that colds were 0.93 (SE 0.22) days shorter, which is over eight times that calculated by Chalmers, and highly significant (*p* = 0.01) [110].

The third paper was a review published in *JAMA* by Michael Dykes and Paul Meier (1975). They analyzed selected studies and concluded that there was no convincing evidence that vitamin C has effects on colds [73]. However, they did not calculate the estimates of the effect nor any *p*-values, and many comments in their analysis were misleading. Pauling wrote a manuscript in which he commented upon the review by Dykes and Meier and submitted it to *JAMA*. Pauling stated afterwards that his paper was rejected even after he twice made revisions to meet the suggestions of the referees and the manuscript was finally published in a minor journal [111,112]. The rejection of Pauling’s papers was strange since the readers of *JAMA* were effectively prevented from seeing the other side of an important controversy. There were also other problems that were not pointed out by Pauling; see [1,70].

Although the three papers have serious biases, they have been used singly or in the combinations of two as references in nutritional recommendations, in medical textbooks, in texts on infectious diseases and on nutrition, when the authors claimed that vitamin C had been shown to be ineffective for colds [1] (pp. 21–23, 36–38, 42–45). The American Medical Association, for example, officially stated that “One of the most widely misused vitamins is ascorbic acid. There is no reliable evidence that large doses of ascorbic acid prevent colds or shorten their duration” [113], a statement that was based entirely on Chalmers’s 1975 review. 

These three papers are the most manifest explanation for the collapse in the interest in vitamin C and the common cold after 1975, despite the strong evidence that had emerged by that time that ≥2 g/day vitamin C shortens and alleviates colds [70]. 

## 7. Pneumonia

Pneumonia is the most common severe infection, which is usually caused by bacteria and viruses.

As recounted at the beginning of this review, the association between frank vitamin C deficiency and pneumonia was noted by Alfred Hess and other early authors, when the chemical identity of vitamin C was not yet known. Vitamin C was purified in the early 1930s and soon thereafter a few German and U.S. physicians proposed that vitamin C might be beneficial in the treatment of pneumonia. For example, Gander and Niederberger (1936) concluded from a series of 15 cases that “the general condition is always favorably influenced (by vitamin C) to a noticeable extent, as is the convalescence, which proceeds better and more quickly than in cases of pneumonia, which are not treated with vitamin C” [114] and other German physicians also claimed benefits of vitamin C [115,116]. Translations of these papers are available [9]. Case reports from the USA also suggested that vitamin C was beneficial against pneumonia [117,118,119]. 

A Cochrane review on vitamin C and pneumonia identified three controlled trials that reported the number of pneumonia cases in participants who were administered vitamin C and two therapeutic trials in which pneumonia patients were given vitamin C [27,28]. 

### 7.1. Vitamin C and the Incidence of Pneumonia 

Table 7 shows the findings of the three vitamin C and pneumonia trials. Each of them found a ≥80% lower incidence of pneumonia for their vitamin C group [27,28,120]. 

Glazebrook (1942) studied male students (15–20 years) in a boarding school in Scotland during World War II [97]. No formal placebo was used; however, 0.05 to 0.3 g/day of vitamin C was added to the morning cocoa and to an evening glass of milk in the kitchen. Thus, the placebo effect does not seem to be a relevant concern in the dining hall. The ordinary diet of the schoolboys contained only 0.015 g/day vitamin C so that their intake was particularly low. 

Kimbarowski (1967) studied the effect of 0.3 g/day of vitamin C on military recruits who had been hospitalized because of influenza type-A in the former Soviet Union [121]. Thus, these pneumonia cases were complications of the viral respiratory infection. Vitamin C also shortened the mean stay in hospital for pneumonia treatment (9 vs. 12 days).

The latest of the three pneumonia prevention trials was carried out during a two-month recruit training period with U.S. Marine recruits by Pitt (1979) [122]. The dose of vitamin C was 2 g/day. This was a randomized double-blind placebo-controlled trial, whereas the two earlier studies were not.

The findings of the three studies are consistent with the notion that the level of vitamin C intake may influence the risk of pneumonia. However, all the three studies were carried out using special participants under particular conditions, and their findings cannot be generalized to the ordinary current Western population. Dietary vitamin C intake was particularly low in the oldest study, and may also have been low in the second study. Thus, the benefit of vitamin C supplementation may be explained by the correction of marginal deficiency in these two older studies. However, in the study by Pitt (1979), the baseline plasma level of vitamin C, 57 µmol/L, corresponds to the dietary vitamin C intake of about 0.1 g/day [11]. Furthermore, although the dose of 2 g/day was high, the plasma level of vitamin C increased only by 36% for the vitamin C group. This also indicates that the basal dietary intake vitamin C was high. Thus, treating marginal vitamin C deficiency is not a reasonable explanation for that latest study.

It is also worth noting that two of these trials used military recruits, and the third used young males who were accommodated in a boarding school [123]. Therefore, the exposure to viruses and bacteria causing pneumonia may have been much higher compared to children and young adults living at home. In each of the three trials, the incidence of pneumonia in the control group was very high when compared with the incidence in the ordinary population [124,125]. A high incidence of pneumonia has been reported in military recruits [126], but the incidence of pneumonia has been even higher in some child populations of the developing countries [127] (Table 7).

It seems reasonable to consider that these three studies observed a true effect of vitamin C against pneumonia in their specific circumstances. However, these findings should not be extrapolated to different circumstances. It would seem worthwhile to examine the effect of vitamin C in population groups that have a high incidence of pneumonia concomitantly with a low intake of vitamin C [27,41].

### 7.2. Vitamin C in the Treatment of Pneumonia

Two studies have reported on the therapeutic effect of vitamin C for pneumonia patients [27,28]. 

Hunt (1994) carried out a randomized, double-blind placebo controlled trial with elderly people in the UK (mean age 81 years), who were hospitalized because of acute bronchitis or pneumonia [26]. The mean plasma vitamin C level at baseline was 23 µmol/L and one third of the patients had a vitamin C level of just ≤11 µmol/L. There was a significant difference in the effect of 0.2 g/day of vitamin C between patients who were more ill and those who were less ill when admitted to the hospital. Vitamin C reduced the respiratory symptom score in the more ill patients but not in their less ill counterparts. There were also six deaths during the study, all among the more ill participants: five in the placebo group, but only one in the vitamin C group.

Mochalkin (1970) examined the effect of vitamin C on pneumonia patients in the former Soviet Union [25]. Although a placebo was not administered to the control group, two different doses of vitamin C were used and the observed difference between the low and high dosage cannot be explained by the placebo effect. The high-dose regime administered on average twice the quantity of vitamin C of the low dose, but both of them were related to the dosage of antibiotics so that the low dose vitamin C ranged from 0.25 to 0.8 g/day, and the high dose ranged from 0.5 to 1.6 g/day. The duration of hospital stay in the control group (no vitamin C supplementation) was 23.7 days. In the low dose vitamin C group the hospital stay was 19% shorter and in the high dose vitamin C group it was 36% shorter. A benefit was also reported on the normalization of chest X-ray, temperature, and erythrocyte sedimentation rate. 

Although both of these therapeutic studies give support to the old case reports stating that vitamin C is beneficial for pneumonia patients, the findings cannot be directly generalized to typical pneumonia patients of Western countries.

## 8. Tetanus

Tetanus is a disease caused by the toxin of *Clostridium tetani*, which may contaminate wounds. An early case report claimed that vitamin C was beneficial against tetanus in an unvaccinated six-year-old boy in the USA [128]. A Cochrane review identified one controlled trial in which the effect of vitamin C on tetanus patients was examined [129,130].

Jahan (1984) studied the effect of 1 g/day of intravenous vitamin C on tetanus patients in Bangladesh [131]. In children aged one to 12 years, there were no deaths in the vitamin C group, whereas there were 23 deaths in the control group (*p* = 10^−9^) [1] (p. 17). In tetanus patients aged 13 to 30 years, there were 10 deaths in the vitamin C group compared with 19 deaths in the control group (*p* = 0.03). The significant difference between the above-described age groups may be caused by the difference in the body weights of the patients. In the young children the same dose of vitamin C corresponds to a substantially higher dose per unit of weight. Although there were methodological weaknesses in the trial, they are unlikely explanations for the dramatic difference in the younger participants [129].

## 9. Other Infections

The effect of vitamin C supplementation on the common cold has been most extensively studied. One important reason for extensive research on vitamin C and the common cold seems to be the wide publicity given to it by Pauling [1,132]. Probably some researchers wanted to show that Pauling was either right or wrong, whereas others just wanted to study a topic about which a Nobel Prize winner had put his credibility on the line. Another reason for the large number of studies on the common cold is that it is a non-severe ubiquitous infection, and it is very easy to find common cold patients in schools and work places. It is much more difficult to study more serious infections.

The three infections discussed above, the common cold, pneumonia, and tetanus, were selected on the basis that the effects of vitamin C have been evaluated in Cochrane reviews, which entails a thorough literature search and a careful analysis of the identified trials. However, the selection of these three infections does not imply that the effects of vitamin C are limited to them. 

Table 2 indicates that vitamin C may have effects on various infections caused by viruses, bacteria, *Candida albicans* and protozoa. Vitamin C might have similar effects in humans. However, it also seems evident that the role of additional vitamin C depends on various factors such as the initial dietary intake level, other nutritional status, the exposure level to pathogens, the level of exercise and temperature stress, etc.

Three extensive searches of the older literature on vitamin C and infections have been published, and they give an extensive list of references, but none of these publications gave a balanced discussion of the findings [133,134,135]. A few studies on the possible effects of vitamin C on other infections are outlined below, but this selection is not systematic. 

Terezhalmy (1978) [136] used a double-blind placebo-controlled RCT and found that the duration of pain caused by herpes labialis was shortened by 51%, from 3.5 to 1.3 days (*p* = 10^−8^), when patients were administered 1 g/day of vitamin C together with bioflavonoids [1] (pp. 15–17). Furthermore, when vitamin C treatment was initiated within 24 hours of the onset of the symptoms, only six out of 26 patients (23%) developed herpes vesicles, whereas with later initiation of vitamin C, eight out of 12 patients (67%) developed vesicles (*p* = 0.003 in the test of interaction). Vitamin C was administered with bioflavonoids, so the study was not specific to vitamin C, but there is no compelling evidence to indicate that bioflavonoids affect infections.

Herpes zoster (reactivation of varicella zoster virus) can cause long lasting post-herpetic neuralgia (PHN). Chen (2009) found that patients with PHN had significantly lower plasma vitamin C plasma than healthy volunteers, and their RCT showed that vitamin C administration significantly decreased the pain level of PHN [137]. A number of other reports have also suggested that vitamin C may be effective against the pain caused by herpes zoster [138,139,140,141,142].

Patrone (1982) and Levy (1996) reported that vitamin C administration was beneficial to patients who had recurrent infections, mainly of the skin [143,144]. Many of the patients had impaired neutrophil functions and therefore the findings cannot be generalized to the ordinary population.

Galley (1997) reported that vitamin C increased the cardiac index in patients with septic shock [145]. Pleiner (2002) reported that intravenous vitamin C administration preserved vascular reactivity to acetylcholine in study participants who had been experimentally administered *Escherichia coli* endotoxin [146]. 

It seems unlikely that the effects of vitamin C on herpetic pain, cardiac index and the vascular system are mediated through effects on the immune system. Such effects are probably caused by other mechanisms instead. The question of the possible benefits of vitamin C against infections is therefore not just a question about the immune system effects of the vitamin, as was discussed earlier in this review (see Section 2.7).

Some physicians used vitamin C for a large set of infectious disease patients and described their experiences in case reports that are worth reading [89,147].

## 10. Observational Studies on Vitamin C and Infections

Cohort studies on vitamins are often unreliable because diet is strongly associated with numerous lifestyle factors that cannot be fully adjusted for in statistical models. Therefore, there may always remain an unknown level of residual confounding [148]. The main source of vitamin C in the diet is fruit, and high dietary vitamin C intake essentially always means a high fruit intake [149]. Thus, any substantial correlations between vitamin C intake and infections could also reflect some other substances in fruit. Only two observational studies are commented upon in this section.

Merchant (2004) studied men whose ages ranged from 40 to 75 years in the USA and found no association between their vitamin C intake and community-acquired pneumonia [124]. These males were U.S. health professionals; thus they were of a population that has a great interest in factors that affect health. The incidence of pneumonia was only three cases per 1000 person-years (Table 7). The median vitamin C intake of the lowest quintile was 0.095 g/day and of the highest quintile it was 1.1 g/day. In contrast, the overall median of the adult U.S. population is about 0.1 g/day, and 10% of the U.S. population has an intake level of less than 0.04 g/day [14]. Thus, Merchant and colleagues’ cohort study indicates that increasing the vitamin C intake upwards from the median level in the USA will not lead to any further decline in the already low pneumonia incidence among male health professionals. However, the study is uninformative about whether decreasing vitamin C level downwards from 0.1 g/day might increase pneumonia risk, or about whether vitamin C might have effects in populations that have particularly high incidences of pneumonia (Table 7). Even though we must be cautious about interpreting observational studies, it seems that biological differences, rather than methodological differences, are most reasonable explanations for the divergence between the findings in the Merchant et al. cohort study and the three controlled trials shown in Table 7.

A cohort analysis of Finnish male smokers that is part of the Alpha-Tocopherol Beta-Carotene Cancer prevention (ATBC) Study found a significant inverse association between dietary vitamin C intake and tuberculosis risk in participants who were not administered vitamin E supplements [150,151]. The highest quartile had the median dietary vitamin C intake level of 0.15 g/day, whereas the lowest quartile had an intake level of only 0.052 g/day. The adjusted risk of tuberculosis in the lowest vitamin C intake quartile was 150% higher than that of the highest intake quartile. This is consistent with the animal studies that found that low vitamin C intake increases the susceptibility to, and severity of, tuberculosis (Table 1, Table 2 and Table 3). 

## 11. Potentially Harmful Interactions between Vitamins C and E

Vitamin C and vitamin E are both antioxidants and they protect against ROS. Therefore, these substances are of parallel interest as water-soluble vitamin C regenerates the lipid-soluble vitamin E in vitro [152]. Dietary vitamin C intake modified the effect of vitamin E on mortality in the ATBC Study, which indicates that these substances may also have clinically important interactions [153]. However, the major sources of the vitamin C in this subgroup were fruit, vegetables and berries and other substances in these foods might also have explained the modification of the vitamin E effect. Such a possibility was refuted by calculating the residual intake of fruit, vegetables and berries, and showing that the residual did not modify the effect of vitamin E. Vitamin C was thus indicated as the specific modifying factor. A similar approach was used to show that vitamin C specifically modified the effect of vitamin E on pneumonia [154].

Two subgroups of the ATBC Study were identified in which the combination of high dietary vitamin C intake and vitamin E supplementation increased the risk of pneumonia by 248% and 1350% when compared with high vitamin C intake without vitamin E (Table 8). In the former subgroup, one extra case of pneumonia was caused for every 13 participants and in the latter subgroup, for every 28 participants. In both subgroups, the residual intake of fruit, vegetables and berries did not modify the effect of vitamin E, indicating specificity of vitamin C. The total number of participants in the ATBC Study was 29,133 and in that respect the identified subgroups were relatively small and at 1081 individuals only amounted to 4% of all the ATBC participants. However, in these two subgroups the harm arising from the combination of vitamins C and E was substantial [154].

Another subgroup analysis of the ATBC Study found that the combination of high vitamin C intake together with vitamin E supplementation increased the risk of tuberculosis in heavy smokers by 125% compared with high vitamin C alone subgroup (Table 8). Thus, one extra case of tuberculosis arose in every 240 participants who had high intakes of vitamins C and E [150,151]. 

ROS have been implicated in the pathogenesis of diverse diseases, including infections. Antioxidants have been assumed to be beneficial since they react with ROS. However, given the suggestions that people should take vitamins C and E to improve their immune system, the subgroup findings in Table 8 are somewhat alarming. Nevertheless, the harm in the three subgroups is limited to the combination of vitamins C and E. This author does not know of any findings that indicate that similar doses of vitamin C alone might cause harm.

## 12. Misconceptions and Prejudices about Vitamin C and Infections

In the first half of the 20th century, a large number of papers were published in the medical literature on vitamin C and infections and several physicians were enthusiastic about vitamin C. The topic was not dismissed because of large-scale controlled trials showing that vitamin C was ineffective. Instead, many rather large trials found benefits of vitamin C. There seem to be four particular reasons why the interest in vitamin C and infections disappeared.

First, antibiotics were introduced in the mid-20th century. They have specific and sometimes very dramatic effects on bacterial infections and therefore are much more rational first line drugs for patients with serious infections than vitamin C. Secondly, vitamin C was identified as the explanation for scurvy, which was considered a disease of the connective tissues. Evidently it seemed irrational to consider that a substance that “only” participates in collagen metabolism might also have effects on infections. However, the biochemistry and actions of vitamin C are complex and not limited to collagen metabolism. Thirdly, the three papers published in 1975 appeared to herald the loss of interest in vitamin C and the common cold (Figure 1) and it seems likely that they increased the negative attitude towards vitamin C for other infections as well. Fourthly, “if a treatment bypasses the medical establishment and is sold directly to the public ... the temptation in the medical community is to accept uncritically the first bad news that comes along” [155]. 

The belief that vitamin C is “ineffective” has been widely spread. For example, a survey of general practitioners in the Netherlands revealed that 47% of respondents considered that homeopathy is efficacious for the treatment of the common cold, whereas only 20% of those respondents considered that vitamin C was [156]. Prejudices against vitamin C are not limited to the common cold. Richards compared the attitudes and arguments of physicians to three putative cancer medicines, 5-fluorouracil, interferon and vitamin C, and documented unambiguous bias against vitamin C [157,158,159]. Goodwin and Tangum gave several examples to support the conclusion that there has been a systematic bias against the concept that vitamins may yield benefits in levels higher than the minimum needed to avoid the classic deficiency diseases [160]. 

The use of vitamin C for preventing and treating colds falls into the category of alternative medicine under the classifications used by the National Institutes of Health in the USA and of the Cochrane collaboration. However, such categorization does not reflect the level of evidence for vitamin C, but reflects the low level of acceptance amongst the medical community, and may further amplify the inertia and prejudices against vitamin C [161].

## 13. Conclusions

From a large series of animal studies we may conclude that vitamin C plays a role in preventing, shortening, and alleviating diverse infections. It seems evident that vitamin C has similar effects in humans. Controlled studies have shown that vitamin C shortens and alleviates the common cold and prevents colds under specific conditions and in restricted population subgroups. Five controlled trials found significant effects of vitamin C against pneumonia. There is some evidence that vitamin C may also have effects on other infections, but there is a paucity of such data. The practical importance and optimally efficacious doses of vitamin C for preventing and treating infections are unknown. Vitamin C is safe and costs only pennies per gram, and therefore even modest effects may be worth exploiting.

## Figures and Tables

**Table 1:** Effect of vitamin-C-rich foods on infections in guinea pigs.

Infection	No. of Studies	No. of Studies with Benefit in Any Infectious Disease Outcome with *p* ≤ 0.01
All	28	20
Tuberculosis (TB)	11	7
Bacterial infection (non-TB) ^a^	15	11
Diphtheria toxin	2	2

One group of guinea pigs was administered a vitamin-C-poor diet, and the other group was administered oranges, cabbage, etc. as supplements to the vitamin-C-poor diet. Based on Appendix 3 in Hemilä (2006) [1] (pp. 119–121). See Supplementary file 1 of this review for the list of the studies. *p*(1-tail) is used in this table. ^a^ Bacterial infections included pneumococcus, group C streptococcus, *Staphylococcus*, and *Salmonella typhimurium.*

**Table 2:** Effect of pure vitamin C on infectious disease outcomes in animal studies.

Category	No. of Studies in the Category	No. of Studies with Benefit in Any Infectious Disease Outcome with *p* ≤ 0.01
All studies	148	86
Time of publication		
Published in 1935–1949	40	20
Published in 1950–1989	48	32
Published in 1990–2005	60	34
Animal species		
Monkey	13	4
Guinea pig	36	21
Cow, sheep, rabbit	10	8
Cat	1	1
Rat	15	10
Gerbil, hamster	7	5
Mouse	18	9
Mammals ^a^	100	58
Birds	13	8
Fish	35	20
Etiological agent		
Tuberculosis (TB)	8	3
Bacteria (non-TB)	70	36
Bacterial toxins	19	16
Virus	22	12
*Candida albicans*	6	4
Protozoa	23	15

A shorter version of this table was published in Hemilä (2006) [1] (p. 8). This table is based on data collected and analyzed in Appendix 2 of [1] (pp. 105–118). See Supplementary file 1 of this review for the list of the studies and their characteristics. *p*(1-tail) is used in this table. ^a^ The mammals category combines all the mammal species from the rows above.

**Table 3:** Infectious agents in studies in which vitamin C decreased the mortality of mammals by *p* ≤ 0.025.

All Studies	29
Tuberculosis (TB)	6
Bacteria (non-TB) ^a^	7
Bacterial toxin ^b^	6
Virus (rabies)	1
*Candida albicans*	2
Protozoa ^c^	7

Table 3 is restricted to mortality as the outcome, and to studies in which the effect of vitamin C on mortality was statistically significant. See Supplementary file 1 of this review for a list of the studies in which vitamin C decreased mortality by *p* ≤ 0.025 (1-tail). In comparison, Table 2 includes studies with all infectious disease outcomes, such as incidence without the animals dying, and various forms of severity of infectious diseases. ^a^ Bacterial infections included pneumococcus and β-hemolytic streptococci; ^b^ Bacterial toxins included diphtheria toxin, tetanus toxin, endotoxin, and a set of clostridial toxins; ^c^ Protozoa infections include *Entamoeba histolytica*, *Leishmania donovani*, *Toxoplasma gondii*, and *Trypanosoma brucei.*

**Table 4:** Effects of regular vitamin C on the incidence and duration of the common cold ^a^.

Outcome Participants	No. of Studies	No. of Participants	Effect of Vitamin C (95% CI)	*p*
Incidence of colds ^b^				
General population	24	10,708	−3% (−6% to 0%)	
People under heavy short-term physical stress	5	598	−52% (−65% to −36%)	10^−6^
Duration of colds		No. of colds		
All studies (≥0.2 g/day)	31	9745	−9.4% (−13% to −6%)	10^−7^
Adults (≥1 g/day)	13	7095	−8% (−12% to −4%)	10^−4^
Children (≥1 g/day)	10	1532	−18% (−27% to −9%)	10^−5^
Severity of colds		No. of colds		
All studies	16	7209	−0.12 (−0.17 to −0.07) ^c^	10^−6^

This table summarizes the main findings of the Cochrane review by Hemilä and Chalker (2013) [68,69]. ^a^ Regular supplementation of vitamin C means that vitamin C was administered each day over the whole study period. Duration and severity of colds indicates the effects on colds that occurred during the study; ^b^ Incidence indicates here the number of participants who had ≥1 cold during the study; ^c^ The unit in this comparison is the standard deviation. Thus −0.12 means that symptoms were decreased by 0.12 times the SD of the outcome.

**Table 5:** Possible differences in the effects of vitamin C on the common cold between subgroups.

Study	Subgroup	Effect of Vitamin C	Outcome	Test of Subgroup Differences (*p*)
Anderson (1972) [80]	Contact with young children	−46%	total days confined to house	0.036
	No contact with young children	−17%		
Anderson (1972) [80]	Usually ≥2 colds per winter	−43%	total days confined to house	0.033
	Usually 0–1 colds per winter	−13%		
Constantini (2011) [86]	Male adolescent competitive swimmers	−47%	duration of colds	0.003
	Female adolescent competitive swimmers	+16%		
Baird (1979) [78]	Male students in UK	−37%	incidence of colds	0.0001
	Female students in UK	+24%		
Carr (1981) [87]	Twins living separately	−35%	duration of colds	0.035
	Twins living together	+1%		

Calculation of the subgroup differences for the Anderson (1972) and the Carr (1981) studies is described in Supplementary file 2. The interactions in the Constantini (2011) and Baird (1979) trials were calculated in [77,86]. *p*(2-tail) is used in this table.

**Table 6:** Variations in vitamin C dose in the control and vitamin C groups.

	Vitamin C Level (g/Day)		
Trial Country, Participants	Dietary Intake Level in the Control Group	Supplement to the Control Group ^a^	Supplement to the Vitamin C Group
Cowan (1942) [100] USA, schoolchildren	?		0.025–0.05
Baird (1979) [78] UK, students	0.05		0.08
Glazebrook (1942) [97] UK, boarding school boys	0.015		0.05–0.3
Peters (1993) [99] South Africa, marathon runners	0.5		0.6
Sabiston (1974) [98] Canada, military recruits	0.04		1
Carr (1981) [87] Australia, twins	?	0.07	1
Karlowski (1975) [72] USA, NIH employees	^b^		3 6

Modified from Table 12 from Hemilä (2006) [1] (p. 34). ^a^ In addition to Carr (1981), a few studies administered 0.01 to 0.05 g/day of vitamin C to the placebo group, but they are not listed here; ^b^ In the 1970s, the average vitamin C intake in the USA was approximately 0.1 g/day. The participants of the Karlowski (1975) study were employees of the National Institutes of Health and therefore their mean dietary intake of vitamin C probably was higher than the national average, but intake of vitamin C was not estimated.

**Table 7:** Effect of vitamin C on the incidence of pneumonia.

Study	Pneumonia Cases/Total		*p* ^a^	Incidence of Pneumonia in the Control Group (1/1000 Person-Years)
	Vitamin C	Control		
Glazebrook (1942) [97]	0/335	17/1100	0.006	30
Kimbarowski (1967) [121]	2/114	10/112	0.022	9% ^b^
Pitt (1979) [122]	1/331	7/343	0.009	120
		Incidence of pneumonia in selected populations:		
Merchant (2004) [124]		Middle-aged males in the USA		3
Hemilä (2004) [125]		Middle-aged males in Finland		5
Pazzaglia (1983) [126]		Military recruits in the USA		60
Paynter (2010) [127]		Children in developing countries, up to		400

Modified from Hemilä (2006) [1] (p. 51). ^a^ Mid-*p* (1-tail); combined test for all three sets of data: *p* = 0.00002 [120]; ^b^ 9% of the hospitalized influenza A patients contracted pneumonia.

**Table 8:** Increase in pneumonia and tuberculosis risk with the combination of vitamins C and E.

Infection, ATBC Study Subgroup	No. of Participants	Effect of Vitamin E RR (95% CI)	Test of Interaction *p*	NNH
Pneumonia				
Body weight < 60 kg who started smoking at ≤20 years				
Dietary vitamin C				
<median	467	0.98 (0.48 to 2.0)	0.026	
≥median (75 mg/day)	468	3.48 (1.61 to 7.5)		13
Pneumonia				
Body weight ≥ 100 kg who started smoking at ≤20 years				
Dietary vitamin C				
<median	613	1.37 (0.46 to 4.0)	0.019	
≥median (95 mg/day)	613	14.5 (1.84 to 114)		28
Tuberculosis				
Smoking ≥ 20 cigarettes/day				
Dietary vitamin C				
<median	9073	0.82 (0.50 to 1.33)	0.011 ^a^	
≥median (90 mg/day)	8172	2.25 (1.19 to 4.23)		240

Subgroups of the ATBC Study in which vitamin C increased the risk of pneumonia and tuberculosis [150,151,154]. ATBC Study, Alpha-Tocopherol Beta-Carotene Cancer prevention Study. NNH, number needed to harm: how many people in the particular subgroup need to be exposed to the treatment to cause harm to one person. RR, relative risk. ^a^ Interaction test was calculated for this review.

## Supplementary Materials

The following are available online at http://www.mdpi.com/2072-6643/9/4/339/s1, Supplementary file 1 and Supplementary file 2.
