# Supplementary material for: Vitamin C and Infections
Source: Nutrients. 2017 Mar 29;9(4):339. doi: 10.3390/nu9040339 (PMC5409678; doi:10.3390/nu9040339)
Supplement: Supplementary file 1 [file nutrients-09-00339-s001.zip › Hemila_Nutrients_Supplement_2.pdf]

Vitamin C and infections.

Harri Hemilä

Supplementary file 2
2017-3-19

Nutrients
http://www.mdpi.com/journal/nutrients

Harri Hemilä, MD, PhD
Department of Public Health,
POB 20 University of Helsinki
Tukholmankatu 8 B
FIN-00014 Helsinki
Finland
harri.hemila@helsinki.fi
http://www.mv.helsinki.fi/home/hemila

Contents

Page

2. Dose-response analysis of the Karlowski (1975) trial
3. Dose-response analysis of the Anderson (1974) trial
4. Table 5. Subgroup comparison of the Anderson (1972) trial by contact with children.
5. Table 5. Subgroup comparison of the Anderson (1972) trial by usual colds.
6. Table 5. Subgroup comparison of the Carr (1981) trial by living together or living apart.
7. Calculation of the ratio for common cold duration SD per mean for imputations

Dose-response analysis of the Karlowski (1975) trial

The main publication of Karlowski (1975) was in JAMA:
https://www.ncbi.nlm.nih.gov/pubmed/163386
The SE values for the four groups were published in a parallel publication (Table 1):
https://www.ncbi.nlm.nih.gov/pubmed/1106302
https://doi.org/10.1111/j.1749-6632.1975.tb29309.x

vitamin C g/day	Group	Karlowski (1975)	Effect in %	No of colds	SE	Var	Weight for linear regression<br>Inverse Var
0	#0	7.14	0.00%	65	0.46	0.21	4.726
3	#1	6.46	-9.52%	56	0.39	0.15	6.575
3	#2	6.71	-6.02%	52	0.53	0.28	3.560
6	#3	5.92	-17.09%	76	0.40	0.16	6.250

Calculation of dose response using the inverse variance weight:
Change indicates the effect of vitamin C in percentages.

```
> Karlowski
   Change InvVar Dose vitC
1   0.000  4.726    0    0
2  -9.524  6.575    3    1
3  -6.022  3.560    3    1
4 -17.087  6.250    6    1

> Karlowski0  <- lm(Karlowski$Change ~ Karlowski$Dose, weights = Karlowski$InvVar)
> summary(Karlowski0)

Coefficients:
                 Estimate Std. Error t value Pr(>|t|)
(Intercept)       0.1379     1.4812   0.093   0.9343
Karlowski$Dose   -2.8534     0.3827  -7.456   0.0175 *

Residual standard error: 3.785 on 2 degrees of freedom
Multiple R-squared:  0.9653,    Adjusted R-squared:  0.9479
F-statistic: 55.59 on 1 and 2 DF,  p-value: 0.01752
```

Comparison of **uniform** vitamin C effect versus **uniform + linear** vitamin C effect:
Inclusion of the linear effect improves the statistical model by Chisq (1 df) = 9.78, p = 0.0017.

```
> Karlowski1  <- lm(Karlowski$Change ~ Karlowski$vitC, weights = Karlowski$InvVar)
> Karlowski2  <- lm(Karlowski$Change ~ Karlowski$vitC + Karlowski$Dose, weights = Karlowski$InvVar)

> lrtest(Karlowski1,Karlowski2)
Likelihood ratio test

Model 1: Karlowski$Change ~ Karlowski$vitC
Model 2: Karlowski$Change ~ Karlowski$vitC + Karlowski$Dose
  #Df   LogLik Df  Chisq Pr(>Chisq)
1   3 -11.2152
2   4  -6.3212  1 9.7879   0.001757
---
```

Dose-response analysis of the Anderson (1974) trial

The Anderson (1974) data were published (Table II)

Placebo group #4 is for reasons described on p. 40 (Table 16) of Hemilä (2006) [1].
Table II gives the mean duration of colds as follows:

vitamin C g/day	Group	Reported<br>Total no. colds	Reported<br>Total days in group	Calculated<br>Days per episode	Imputed<br>SD	SE	Var	Weight for linear regression<br>Inverse Var	Effect in %
0	#4	437	1539	3.522	3.205	0.15	0.0235	42.549	0.00%
4	#7	417	1325	3.177	2.891	0.14	0.0200	49.876	-9.78%
8	#8	483	1380	2.857	2.600	0.12	0.0140	71.450	-18.87%
					SD= 0.91*mean				

Anderson (1974) did not publish the SD for the observations.
Above, SD is imputed with ratio SD = 0.91 * mean. See the last page of this document.
Furthermore, in Anderson (1972) the SD per mean ratio was 0.97 and 0.82 in the vitamin C and placebo groups, see the last page. Thus, the ratio 0.92 is also close to the ratios of Anderson's earlier study.

```
> A1974
  Change InvVar Dose vitC
1   0.00 42.549    0    0
2  -9.78 49.876    4    1
3 -18.87 71.450    8    1

> Anderson0  <- lm(A1974$Change ~  A1974$Dose, weights = A1974$InvVar)
> summary(Anderson0)

Coefficients:
              Estimate Std. Error t value Pr(>|t|)
(Intercept) -0.13778    0.27584   -0.50    0.705
A1974$Dose  -2.35178    0.04818  -48.81    0.013 *


Residual standard error: 2.011 on 1 degrees of freedom
Multiple R-squared:  0.9996,    Adjusted R-squared:  0.9992
F-statistic:  2382 on 1 and 1 DF,  p-value: 0.01304
```

Table 5. Subgroup comparison of
the Anderson (1972) trial by contact with children.
https://www.ncbi.nlm.nih.gov/pmc/articles/PMC1940935

The ratio between SD and mean was calculated from the overall results of the trial
Anderson (1972) reported that (Table II):

Vitamin C group
Among 407 participants, for “days confined to house” SE = 0.101, corresponding to SD = 2.038.
The mean “days confined to house” was 1.30 so that the ratio = 1.567 (= 2.038/1.30)
Placebo group
Among 411 participants, for “days confined to house” SE = 0.138, corresponding to SD = 2.798.
The mean “days confined to house” was 1.87 so that the ratio = 1.496 (= 2.798/1.87)

The ratio of 1.57 was used for the imputation of the SD from the subgroup means.

Anderson (1972) reported that (Tables I and IV):

**Contact with young children**	**Vitamin C**	**Placebo**	**Effect of vitamin C**
**Yes**			
Days confined to house:	1.31	2.43	-46.1%
Imputed SD:	2.06	3.82	
No of participants:	140	148	
**No**			
Days confined to house:	1.30	1.56	-16.7%
Imputed SD:	2.04	2.45	
No of participants:	267	263	

	Vitamin C			Placebo				Mean Difference	Mean Difference
**Study or Subgroup**	**Mean**	**SD**	**Total**	**Mean**	**SD**	**Total**	**Weight**	**IV, Fixed, 95% CI**	**IV, Fixed, 95% CI**
**1.1.1 Contact young children**									
Anderson 1972	1.31	2.06	140	2.43	3.82	148	23.0%	-1.12 [-1.82, -0.42]	
**Subtotal (95% CI)**			**140**			**148**	**23.0%**	**-1.12 [-1.82, -0.42]**	
Heterogeneity: Not applicable									
Test for overall effect: Z = 3.12 (P = 0.002)									
**1.1.2 No contact young children**									
Anderson 1972	1.3	2.04	267	1.56	2.45	263	77.0%	-0.26 [-0.64, 0.12]	
**Subtotal (95% CI)**			**267**			**263**	**77.0%**	**-0.26 [-0.64, 0.12]**	
Heterogeneity: Not applicable									
Test for overall effect: Z = 1.33 (P = 0.18)									
**Total (95% CI)**			**407**			**411**	**100.0%**	**-0.46 [-0.79, -0.12]**	
Heterogeneity: Chi² = 4.42, df = 1 (P = 0.04); I² = 77%									
Test for overall effect: Z = 2.66 (P = 0.008)									
Test for subgroup differences: Chi² = 4.42, df = 1 (P = 0.04), I² = 77.4%									

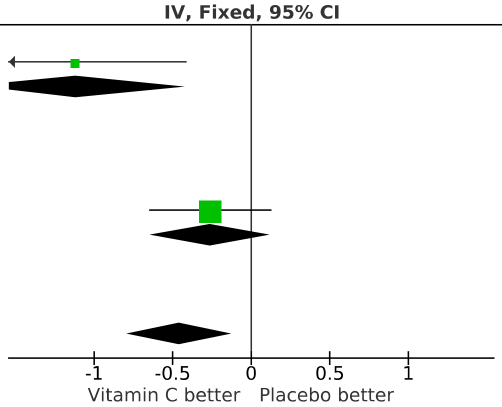

Table 5. Subgroup comparison of the Anderson (1972) trial by usual colds.

Anderson (1972) reported that (Tables I and IV):

The ratio 1.6 was used in the imputation of the SD for the means of the subgroups, see above.

**Usual colds per winter**	**Vitamin C**	**Placebo**	**Effect of vitamin C**
**≥2 colds**			
Days confined to house:	1.31	2.29	-42.8%
Imputed SD:	2.06	3.60	
No of participants:	211	192	
**0-1 colds**			
Days confined to house:	1.30	1.50	-13.3%
Imputed SD:	2.04	2.36	
No of participants:	196	219	

Study or Subgroup	Vitamin C Mean	Vitamin C SD	Vitamin C Total	Placebo Mean	Placebo SD	Placebo Total	Weight	Mean Difference IV, Fixed, 95% CI
**1.2.1 Usually ≥ 2 colds per winter**								
Anderson 1972	1.31	2.06	211	2.29	3.6	192	34.8%	-0.98 [-1.56, -0.40]
**Subtotal (95% CI)**			**211**			**192**	**34.8%**	**-0.98 [-1.56, -0.40]**
Heterogeneity: Not applicable								
Test for overall effect: Z = 3.31 (P = 0.0009)								
**1.2.2 Usually 0-1 colds per winter**								
Anderson 1972	1.3	2.04	196	1.5	2.36	219	65.2%	-0.20 [-0.62, 0.22]
**Subtotal (95% CI)**			**196**			**219**	**65.2%**	**-0.20 [-0.62, 0.22]**
Heterogeneity: Not applicable								
Test for overall effect: Z = 0.93 (P = 0.35)								
**Total (95% CI)**			**407**			**411**	**100.0%**	**-0.47 [-0.81, -0.13]**
Heterogeneity: Chi² = 4.53, df = 1 (P = 0.03); I² = 78%								
Test for overall effect: Z = 2.70 (P = 0.007)								
Test for subgroup differences: Chi² = 4.53, df = 1 (P = 0.03), I² = 77.9%								

Mean Difference
IV, Fixed, 95% CI

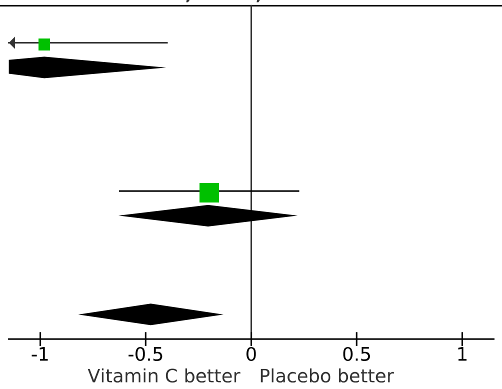

Table 5. Subgroup comparison of
the Carr (1981) trial by living together or living apart.
https://www.ncbi.nlm.nih.gov/pubmed/7048833

Carr (1981) reported that (Table 2):

	**Vitamin C**	**Placebo**	**Effect of vitamin C**
**Living apart**			
Mean duration of colds:	4.86	7.50	-35.2%
No of colds:	94	71	
**Living together**			
Mean duration of colds:	5.46	5.42	+0.7%
No of colds:	57	70	

SD for the duration of colds among twins living apart was calculated from the reported P < 0.01. Assuming P =0.009 corresponds to t(127 df) = 2.62, which corresponds to SD 5.68 days. That SD value was used for both the vitamin C and placebo groups of twins living apart.

SD for twins living together was estimated from the relation that the ratio of SD/mean = 0.91 covers 80% of the reported ratios so that this ratio is conservative, see the last page. Thus SD = 4.9 days(=0.91*5.4 days) was used as the imputed SD for twins living together.

The test for subgroup differences Chi^2 (1 df)= 4.44 corresponds to P = 0.035.

	Vitamin C			Placebo				Mean Difference
**Study or Subgroup**	**Mean**	**SD**	**Total**	**Mean**	**SD**	**Total**	**Weight**	**IV, Fixed, 95% CI**
**1.3.1 Apart**								
Carr 1981a	4.86	5.68	57	7.5	5.68	71	37.0%	-2.64 [-4.62, -0.66]
**Subtotal (95% CI)**			**57**			**71**	**37.0%**	**-2.64 [-4.62, -0.66]**
Heterogeneity: Not applicable								
Test for overall effect: Z = 2.61 (P = 0.009)								
**1.3.2 Together**								
Carr 1981b	5.46	4.9	94	5.42	4.9	70	63.0%	0.04 [-1.48, 1.56]
**Subtotal (95% CI)**			**94**			**70**	**63.0%**	**0.04 [-1.48, 1.56]**
Heterogeneity: Not applicable								
Test for overall effect: Z = 0.05 (P = 0.96)								
**Total (95% CI)**			**151**			**141**	**100.0%**	**-0.95 [-2.15, 0.25]**
Heterogeneity: Chi^2 = 4.44, df = 1 (P = 0.04); I^2 = 77%								
Test for overall effect: Z = 1.55 (P = 0.12)								
Test for subgroup differences: Chi^2 = 4.44, df = 1 (P = 0.04), I^2 = 77.5%								

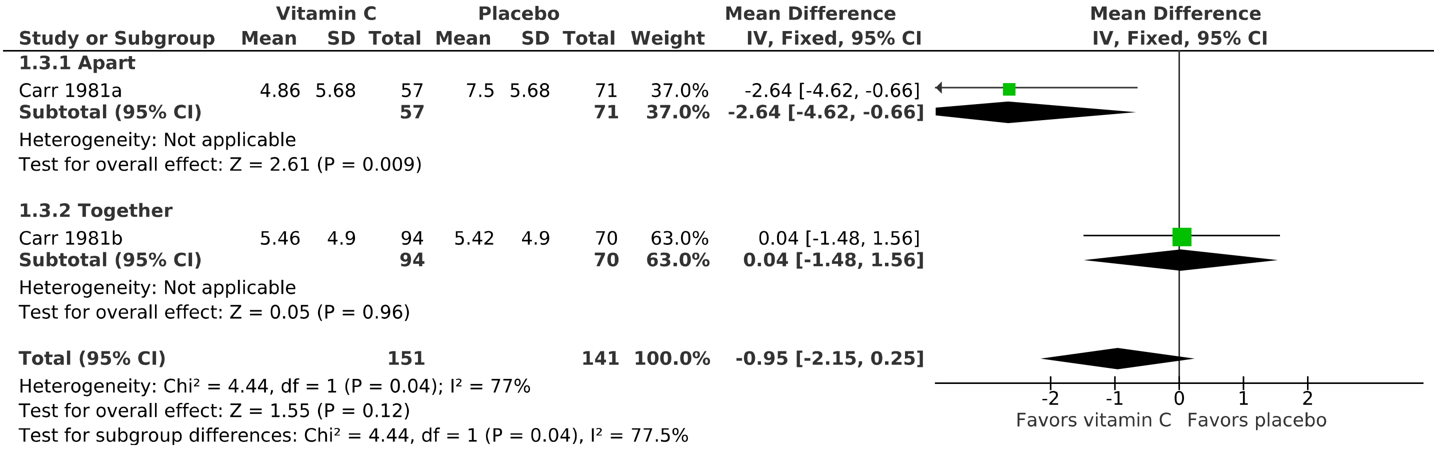

Calculation of the ratio for common cold duration SD per mean for imputations

The imputation of SD was based on the analysis of 67 vitamin C and zinc lozenge study groups for which SD and mean were reported, see below.
The median for the ratio of SD/mean was 0.58 and the 80th percentile was 0.91.
The latter ratio **0.91** was used as the ratio to impute the SD value for the Carr (1981) trial.
The vitamin C trials are listed in Hemilä and Chalker (2013)
https://www.ncbi.nlm.nih.gov/pubmed/23440782
http://www.mv.helsinki.fi/home/hemila/CC
and the zinc lozenge trials are listed in Hemilä (2011)
https://www.ncbi.nlm.nih.gov/pmc/articles/PMC3136969

	**Treatment groups**			**Placebo groups**		
	**Duration in days**			**Duration in days**		
**Vitamin C trials**	**mean**	**SD**	**Ratio**	**mean**	**SD**	**Ratio**
**Prevention trials**						
Anderson-1972	3.96	3.84	**0.97**	4.18	3.43	**0.82**
Charleston-1972	3.55	1.93	**0.55**	4.21	0.82	**0.20**
Clegg-1975	7.2	3.1	**0.43**	7.6	3	**0.39**
Elwood-1976	5.97	5.73	**0.96**	6.38	6.33	**0.99**
Himmelstein 1998sed	2.5	1.1	**0.44**	4.2	3.5	**0.83**
Johnston 2014	2.2	1.4	**0.64**	5.4	4.5	**0.83**
Karlowski 1975a (#2)	6.71	3.82	**0.57**	7.14	3.71	**0.52**
Karlowski 1975d (#3 vs #1)	5.92	3.49	**0.59**	6.46	2.92	**0.45**
Van Straten-2002	1.8	2.98	**1.66**	3.1	4.65	**1.50**
Peters-1993 Runners	6	0.99	**0.17**	5.8	2.46	**0.42**
Peters 1993a Sedentary	4.2	2.14	**0.51**	5.6	3.18	**0.57**
Sabiston-1974	4.3	3	**0.70**	6	3	**0.50**
Bancalari 1984	3.4	2.77	**0.82**	4.5	2.92	**0.65**
Constantini 2011 Male	5.5	5	**0.91**	10.4	7.1	**0.68**
Constantini 2011a Female	8.6	5.5	**0.64**	7.4	8.2	**1.11**
Ludvigsson-1977 Pilot	8.9	5.96	**0.67**	14.53	9.75	**0.67**
Ludvigsson-1977a Large	9.54	8.65	**0.91**	10.14	11.6	**1.14**
Ritzel-1961	1.8	1.29	**0.71**	2.6	1.29	**0.49**
Wilson_1973 Girls	2.62	3.67	**1.40**	3.1	4.15	**1.34**
Wilson_1973a Boys	2.68	5.01	**1.87**	2.48	3.29	**1.33**
**Treatment trials**						
Audera 2001 (1 g/day)	10.1	7	**0.69**	8.5	6.67	**0.79**
Audera 2001a (3 g/day)	10.4	6.67	**0.64**			
Elwood 1977	5.89	2.77	**0.47**	6.29	2.78	**0.44**
Tyrrell 1977 (Males)	7.41	3.59	**0.48**	7.57	3.79	**0.50**
Tyrrell 1977a (Females)	8.96	5.3	**0.59**	8.24	4.78	**0.58**
**Zinc lozenge trials**						
Douglas 1987	12.10	9.80	**0.81**	7.70	9.80	**1.27**
Godfrey 1992	4.86	2.70	**0.56**	6.13	2.70	**0.44**
Macknin 1998	9.37	4.81	**0.51**	9.50	4.53	**0.48**
Mossad 1996	5.20	2.83	**0.54**	9.38	5.47	**0.58**
Petrus 1998	5.29	2.57	**0.49**	7.06	3.91	**0.55**
Prasad 2000	4.44	1.56	**0.35**	8.09	1.81	**0.22**
Prasad 2008	4.00	1.04	**0.26**	7.12	1.26	**0.18**
Turner 2000 A	7.41	3.88	**0.52**	7.55	3.96	**0.52**
Turner 2000 B	6.89	3.35	**0.49**			
Turner 2000 C	7.90	4.20	**0.53**			
		Median	**0.58**			
		Mean	**0.69**			
		Count	**67**			

		**Ratio of SD/mea**
		0.17
	**SD/mean**	0.18
	**ratio**	0.20
	**ordered**	0.22
	**to find**	0.26
	**quartiles**	0.35
		0.39
		0.42
		0.43
		0.44
		0.44
		0.44
		0.45
		0.47
		0.48
		0.48
	**1nd quartile**	0.49
		0.49
		0.50
		0.50
		0.50
		0.51
		0.51
		0.52
	**Half of the ratios**	0.52
	**are between**	0.52
	**0.49 and 0.82**	0.53
		0.54
		0.55
		0.55
		0.56
		0.57
	33	0.57
	**Median**	0,580
	33	0.58
		0.59
		0.59
		0.64
		0.64
		0.64
		0.65
		0.67
		0.67
		0.68
		0.69
		0.70
		0.72
		0.79
		0.81
	**3rd quartile**	**0.82**
		0.82
		0.83
		0.83
	**80th percentile**	**0.91**
		0.91
		0.96
		0.97
	Elwood-1976	0.99
	Constantini 2011a Female	1.11
	Ludvigsson-1977a Large	1.14
	Douglas	1.27
**These might be**	Wilson	1.33
**classified as**	Wilson	1.34
**outliers**	Wilson	1.40
	van Straten	1.50
	van Straten	1.66
	Wilson	1.87
	**Total count**	**67**
